# Investigation of a gold quantum dot/plasmonic gold nanoparticle system for improvement of organic solar cells†

**DOI:** 10.1039/c8na00119g

**Published:** 2018-11-08

**Authors:** Sopit Phetsang, Apichat Phengdaam, Chutiparn Lertvachirapaiboon, Ryousuke Ishikawa, Kazunari Shinbo, Keizo Kato, Pitchaya Mungkornasawakul, Kontad Ounnunkad, Akira Baba

**Affiliations:** Graduate School of Science and Technology, Niigata University 8050 Ikarashi-2-nocho, Nishi-ku Niigata 950-2181 Japan ababa@eng.niigata-u.ac.jp; Department of Chemistry and Center of Excellence for Innovation in Chemistry (PERCH-CIC), Faculty of Science, Chiang Mai University Chiang Mai 50200 Thailand kontad.ounnunkad@cmu.ac.th; Department of Chemistry, Faculty of Science, Prince of Songkla University Hat Yai Songkla 90110 Thailand; Environmental Science Program, Faculty of Science, Chiang Mai University Chiang Mai 50200 Thailand; Center of Excellence in Materials Science and Technology, Chiang Mai University Chiang Mai 50200 Thailand

## Abstract

Light management allows enhancement of light harvesting in organic solar cells (OSCs). In this paper, we describe the investigation of OSCs enhanced by the synergistic effect of gold quantum dots (AuQDs) and localized surface plasmons, obtained by blending a AuQD layer and plasmonic gold nanoparticles (AuNPs) in a hole-transport layer (HTL). Different AuQDs emitting blue, green, and red fluorescence were examined in this study. The OSCs were demonstrated to comprise an ITO-coated glass substrate/AuQDs/PEDOT:PSS:AuNPs/P3HT:PCBM/Al structure. The UV-visible spectra, current density *versus* voltage characteristics, impedance spectra, and incident photon-to-current efficiency of the fabricated devices were evaluated. The results showed an enhancement of photovoltaic efficiency achieved as a result of the increase in short-circuit current density (*J*_sc_) and power conversion efficiency (PCE) in comparison with those of the reference OSCs. The best synergistic effect was found with OSCs consisting of a green-emitting AuQD layer and a HTL containing AuNPs, resulting in the highest improvement in PCE of 13.0%. This indicated that the increase in light harvesting in the developed devices was induced by extended light absorption in the UV region resulting from absorption by the AuQD layer and emission of visible fluorescence from the AuQD layer to the photoactive layers. Moreover, the localized surface plasmon effect of AuNPs, which also contributed to an increase in light trapping in the proposed OSCs, was enhanced by the effect of the AuQDs.

## Abstract 2

The performance of organic solar cells was improved by the effect of a synergistic gold quantum dot/plasmonic gold nanoparticle system.

## Graphical abstract

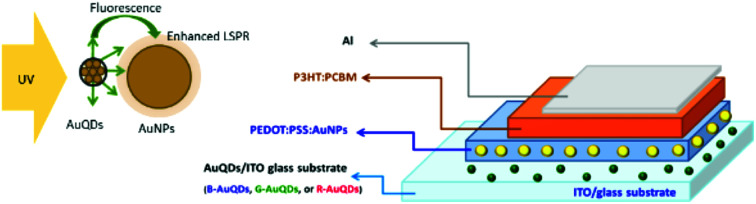

## Introduction

Organic thin-film solar cells (OSCs) are being considered as a promising alternative renewable energy source due to their excellent properties, such as low weight and high flexibility, and the feasibility of low temperature processing, solution-based fabrication, and cost-effective production of OSCs.^1–3^ Although bulk heterojunction (BHJ) OSCs exhibited several advantages and improved the performance of photovoltaic devices, the short carrier diffusion length of polymer materials limits the film thickness of the active layer in the OSCs, resulting in lower power conversion efficiency (PCE) compared with that of traditional solar cells based on crystalline Si.^1,4^ Metallic nanostructure-induced plasmonic properties have been used to improve light harvesting in photovoltaic devices;^5–8^ in particular, the introduction of gold nanoparticles (AuNPs) into OSCs enhanced the photocurrent and PCE.^9–18^ We also reported the use of urchin-like AuNPs to improve the performance of OSCs.^9^ Plasmonic nanoparticles (NPs) are usually dispersed in photoactive or hole-transport layers as well being deposited at the interfaces of organic layers in photovoltaic devices.^19,20^ AuNPs with particle sizes from 2 to 100 nm typically enhance the electric field and optical absorption through excitation of localized surface plasmon resonance, which depends on the particle size, shape, and surrounding environment. When the size of gold nanoparticles is further reduced (<2 nm), they are known as gold nanoclusters or gold quantum dots (AuQDs), on which localized plasmons cannot be excited. Instead, due to the quantum confinement effect, electrons in AuQDs are excited from the ground state by absorbing near-UV light, and the AuQDs emit fluorescence in the visible range. The size of the AuQDs, *i.e.*, the number of gold atoms, determines the wavelength of the fluorescence emission.^21,22^ This implies that AuQDs can harvest light from the UV region and convert it into visible light. AuQDs were previously shown to be effective in the operation of dye-sensitized solar cells, being employed both as a photosensitizer and catalyst. AuQDs deposited on a TiO_2_ electrode improved the light absorption capacity, increased the photocurrent, and enhanced charge transport.^23–27^ Because most organic photoelectric conversion materials harvest light mainly in the visible range, an important challenge is to apply AuQDs to organic light harvesting systems, which absorb light in the near-UV region and convert it to visible light as fluorescence emission.^28^ When combining AuQDs and AuNPs, synergistic effects are expected because the fluorescence from AuQDs enhances the localized plasmon effect of the AuNPs, and simultaneously the enhanced electric field on AuNPs enhances the fluorescence of AuQDs by localized plasmon excitation.^29^

In this work, we demonstrate the synergistic effect of a AuNPs/AuQD system on the OSC performance. The enhancement of the photovoltaic properties of plasmonic OSCs by introducing AuNPs and AuQDs (blue, green, and red) was investigated. AuQDs/plasmonic solar cells with an Al/poly(3-hexylthiophene-2,5-diyl) (P3HT):[6,6]-phenyl C_61_ butyric acid methyl ester (PCBM)/AuNP:poly(3,4-ethylene dioxythiophene):poly(styrene sulfonate) (PEDOT:PSS)/AuQD/indium tin oxide (ITO) glass substrate structure exhibited an improved performance. Our results demonstrated that the AuQDs/AuNPs in the organic solar cells played an important role in improving their photovoltaic properties due to the fluorescence from the AuQDs and energy/electron transfer from the AuQDs to the AuNPs, leading to a 13% increase in the PCE.

## Experimental

### Chemicals and materials

Three types of AuQDs (<2 nm) with different fluorescence emission wavelengths – blue-AuQDs (mixed Au_5_ and Au_8_; 5 and 8 Au atoms), green-AuQDs (Au_13_; 13 Au atoms), and red-AuQDs (Au_25_; 25 Au atoms) (B-AuQDs, G-AuQDs, and R-AuQDs, respectively) – were purchased from Dai Nippon Toryo Co. Ltd. (Japan). All of them were capped by pepsin molecules as a stabilizer.^21^ Poly(3-hexylthiophene-2,5-diyl) (P3HT), [6,6]-phenyl C_61_ butyric acid methyl ester (PCBM), 1,2-dichlorobenzene, and a colloidal solution of AuNPs with an average diameter of 5 nm were purchased from Sigma-Aldrich (Japan). An indium tin oxide (ITO)-coated glass substrate with a conductivity of 10 Ω cm^−2^ was purchased from Furuuchi Chemical (Japan). Concentrated hydrochloric acid (HCl, 37%) and analytical grade acetone were purchased from Sigma-Aldrich (Japan).

### Fabrication of AuQD/plasmonic AuNP-OSCs

The photovoltaic devices were fabricated on ITO-coated glass substrates (area 1.0 cm^2^). After cleaning, the ITO glass substrate was dried and treated with UV/ozone for 20 min to improve the wettability of the ITO surface. To prepare the PEDOT:PSS:AuNP composite buffer layer, a 0.10 mM aqueous solution of AuNPs with a particle size of 5.0 nm was mixed with PEDOT:PSS solution (1 : 6 v/v) by sonicating for 1 h. The aqueous solution of AuQDs (B-AuQDs, G-AuQDs, or R-AuQDs) with an optimized concentration (see Table S1†) was first deposited on the ITO glass substrate by spin-coating and subsequently annealed at 120 °C for 30 min. The PEDOT:PSS:AuNP solution was then spin-coated at 1000 rpm on top of the AuQD layer and annealed at 120 °C for 30 min. The thickness of this layer was approximately 100 nm (ESI, Fig. S1†). A P3HT:PCBM blend solution with a mass ratio of 1 : 0.8 in dichlorobenzene was spin-coated on the PEDOT:PSS:AuNP surface, followed by annealing at 120 °C for 30 min. The P3HT:PCBM functioned as a photoactive layer with a thickness of 100 nm (ESI, Fig. S2†). Finally, a 150 nm thick Al electrode layer was deposited on the P3HT:PCBM layer by thermal evaporation under vacuum. All the devices were annealed at 150 °C for 45 min in a vacuum chamber before further characterization.

### Characterization

The photovoltaic properties and impedance spectra of the fabricated devices were measured with a precision source/measure unit (B2901A, Agilent) and a potentiostat (PARSTAT 4000, Princeton Applied Research), respectively, and the solar cell was operated under illumination from a solar simulator (HAL-C100, a 100 W compact xenon light source, Asahi Spectra) with a light intensity of 75 mW cm^−2^. The UV-visible absorption spectra of the AuQD/PEDOT:PSS:AuNP films and the AuQD/PEDOT:PSS:AuNP/P3HT:PCBM films on ITO glass substrates were evaluated with a UV-vis spectrometer (V-650, Jasco). The surface morphologies of the AuQD films and PEDOT:PSS:AuNP films were characterized using an atomic force microscope (AFM, SPM-9600, Shimadzu, Japan).

## Results and discussion

### Optical properties and surface morphology of the AuQD/plasmonic AuNP systems

**Fig. 1:**
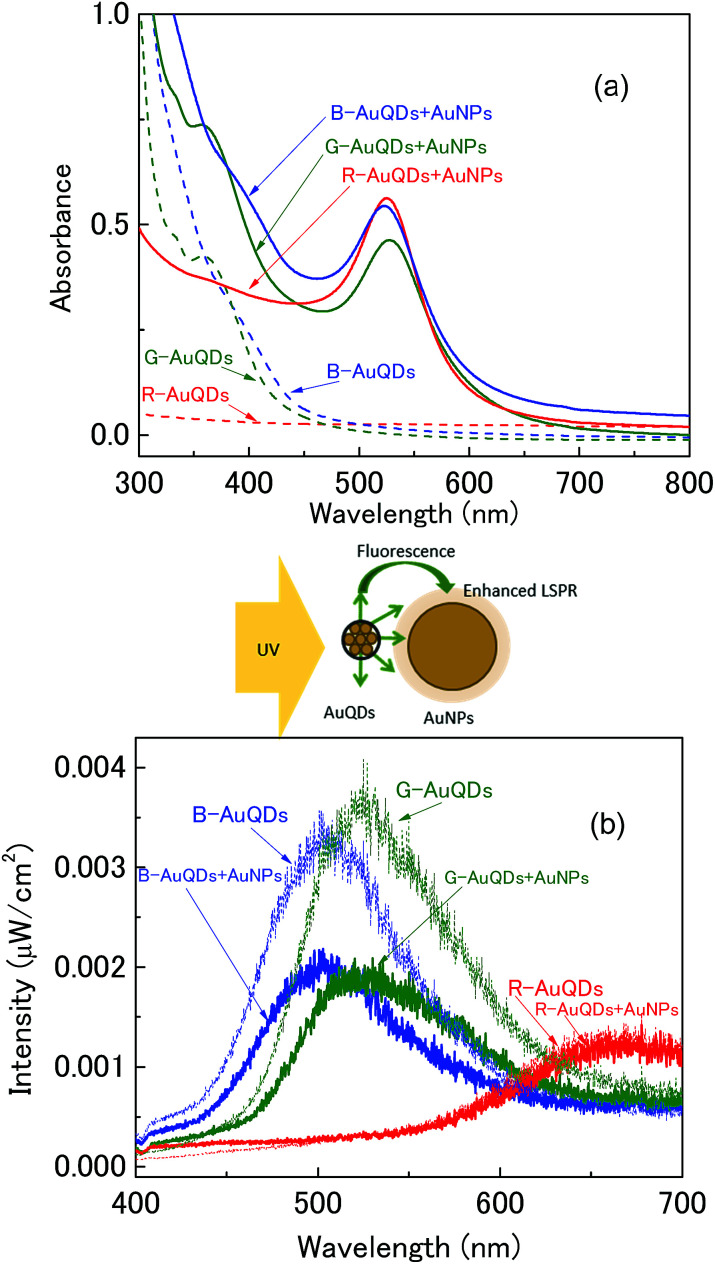
(a) UV-vis absorption spectra and (b) fluorescence spectra of AuQDs and AuQD:AuNP aqueous solution.

Three types of AuQDs – B-AuQDs, G-AuQDs, and R-AuQDs – were used in this study. The emitted visible light from the AuQD layers was expected to increase light trapping by the developed solar cells. The fluorescence of the AuQDs depended significantly on their size, which affected the performance of the plasmonic AuNP solar cells. AuQDs stabilized by pepsin in an aqueous solution exhibit strong absorption at wavelengths below 400 nm and emit fluorescence at longer wavelengths in the visible region.^21^ The absorption spectra of the three AuQD solutions (5.0 μM) are presented in Fig. 1(a) and their fluorescence spectra under UV light illumination (*λ*_ext_ = 350 nm) are shown in Fig. 1(b). In addition, the fluorescence of AuQDs on glass slides was clearly observed with the naked eye under UV light irradiation (ESI, Fig. S3†), where the colors corresponded to those of the solutions. In this measurement, deionized water was added to the AuQDs to maintain the same concentration (5.0 μM) as in the AuQD/AuNP complex system in deionized water, as the added AuNPs were dissolved in deionized water. The absorption spectra of the AuQD/AuNP complex exhibit a localized plasmon peak at around 525 nm in addition to the absorption baseline of AuQDs. It should be noted that the fluorescence peaks of B-AuQDs and G-AuQDs decreased considerably when they were mixed with the AuNPs. This result clearly indicates that the fluorescence emission of AuQDs is quenched by AuNPs. Especially, significant quenching was observed in the G-AuQD/AuNP complex. Because the fluorescence peak of G-AuQDs and the localized plasmon peak overlap significantly, the quenching can be considered mainly as an energy transfer, which should enhance localized plasmon excitation.^24^ This is expected to enhance photocarrier generation in the active layer when the G-AuQD/AuNP complex system is used in OSC devices. However, almost no fluorescence quenching was observed for R-AuQDs/AuNPs. This is reasonable because the overlap between the absorption of AuNPs and the fluorescence emission wavelength of R-AuQDs is very small, resulting in almost no energy transfer in this system.

The surface morphologies of the AuQDs and AuQD/AuNP:PEDOT:PSS films were studied using atomic force microscopy (AFM). In this experiment, an aqueous solution of AuQDs was deposited on an ITO/glass substrate by spin-coating as shown in the ESI, Fig. S4.† The appearance of bright spots on the surface originated from the aggregation of AuQDs with a size of *ca.* 5 nm. Although aggregation of AuQDs was observed on the film surface, a photograph of AuQDs under UV irradiation indicated homogeneous fluorescence (see Fig. S3†), indicating that the non-aggregated AuQDs were uniformly deposited on the substrate together with the aggregated AuQDs. It should be noted that the aggregated AuQDs with a size of 5 nm are expected to exhibit a plasmonic-like effect because the size of Au becomes similar to that of plasmonic AuNPs.^25^ AFM images of PEDOT:PSS films and AuNP:PEDOT:PSS films on AuQD layers are presented in the ESI, Fig. S5.† A similar surface morphology was observed after spin-coating a pristine PEDOT:PSS solution on top of all the AuQD layers (ESI, Fig. S5(a–d)†). In addition, no significant differences in the morphologies of AuNP:PEDOT:PSS films deposited on different AuQD films were observed, and the surfaces exhibited some aggregation of AuNPs with a size of *ca.* 10 nm (ESI, Fig. S5(e–h)†). Because a large degree of aggregation or surface roughness at the interface of the PEDOT:PSS/P3HT:PCBM layer decreases the device performance, we optimized the concentration of AuNPs at 0.1 mM by measuring the OSC efficiency and the AFM morphologies.

### Photovoltaic performance of AuQD/plasmonic AuNP solar cells

**Fig. 2:**
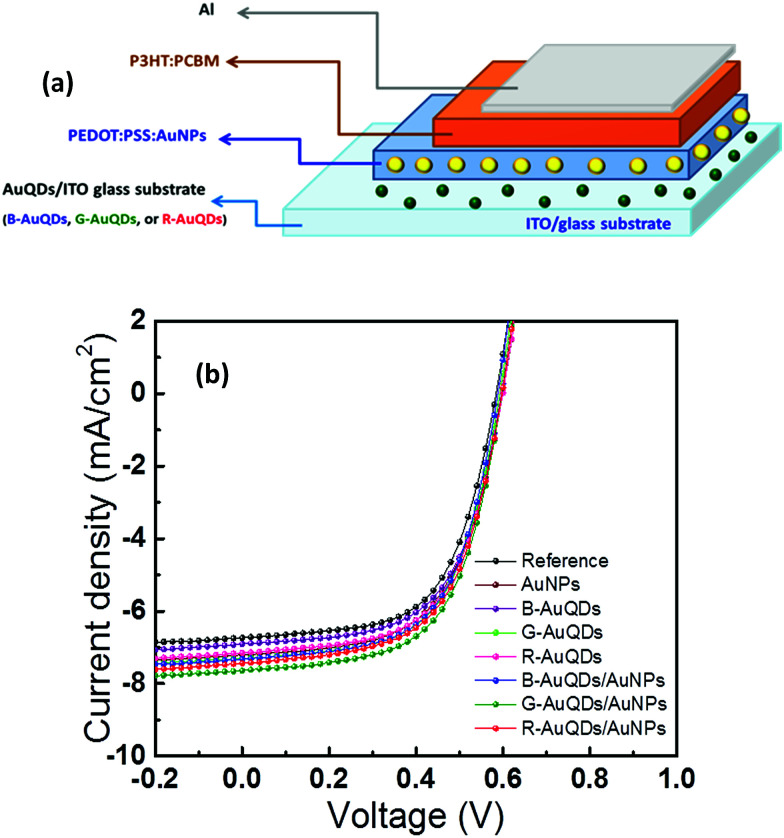
(a) Schematic of the fabricated OSCs and (b) *J*–*V* characteristics of the OSCs compared with that of the reference cell.

The proposed device structure of the AuQD/plasmonic AuNP solar cells consists of Al/P3HT:PCBM/PEDOT:PSS:AuNP/AuQD/ITO glass substrate as shown in Fig. 2(a). AuQDs emitting fluorescence at different wavelengths, B-AuQDs, G-AuQDs, and R-AuQDs, were used to improve the properties of plasmonic AuNP-enhanced photovoltaic devices. Under the same experimental conditions, AuNPs were added to the hole-transport PEDOT:PSS layer for photon trapping, and each AuQD layer was used for UV light harvesting. The current density–voltage (*J*–*V*) characteristics of different AuQD/plasmonic AuNP systems in fabricated solar cells are presented in Fig. 2(b), and the important photovoltaic parameters of the devices are given in Table 1. A device with no metal nanoparticles was used as a reference cell. Specifically, AuNPs enhanced the performance due to a localized surface plasmon effect, which enhanced the optical path lengths *via* light scattering as well as increasing light absorption by the photoactive layer.^5,9,30^ Moreover, a AuQD layer inserted into the OSCs increased the efficiency by absorbing light in the UV region and converting it to visible light^25^ in the cells, in which a higher photogenerated carrier density could be obtained *via* higher absorption of visible light by the active layer. It should be noted that the P3HT:PCBM exhibits almost no photoelectric conversion in the UV region but exhibits absorption peak between 500 and 600 nm. We found that the AuQD/AuNP complex system presented synergistic benefits in light management of the developed OSCs. After incorporating a AuQD layer, B-AuQDs, G-AuQDs, or R-AuQDs, and/or a AuNP:PEDOT:PSS layer into the polymer solar cells, the open-circuit voltage (*V*_oc_) and the fill factor (FF) were found to be similar, while the short-circuit current density (*J*_sc_) increased from 2.92% to 11.1%, and the power conversion efficiency (PCE) increased from 2.47% to 13.0% in comparison with the corresponding values for the reference cell. Of the three AuQDs without added plasmonic AuNPs, incorporating G-AuQDs in the solar cell gave the best performance, with a *J*_sc_ of 7.33 mA cm^−2^ and PCE of 3.50%. Comparative *J*–*V* curves for all the OSCs are clearly shown in the ESI, Fig. S6.† Interestingly, the AuQDs/plasmonic AuNPs embedded in the devices offered improved photovoltaic performance in comparison with only AuQDs or only AuNPs in the OSCs. The results clearly indicated that the *J*_sc_ and PCE of all the AuQD/plasmonic AuNP devices were further enhanced compared to those of the solar cells without plasmonic AuNPs in the HTL. The presence of both a G-AuQD layer and AuNPs in the PEDOT:PSS film produced the greatest increase in the *J*_sc_ from 6.85 to 7.61 mA cm^−2^ and enhanced the PCE from 3.24% to 3.66%, followed by the R-AuQD/plasmonic AuNP- and B-AuQD/plasmonic AuNP-OSCs. The latter two solar cell systems exhibited *J*_sc_ values of 7.38 and 7.20 mA cm^−2^ and PCE values of 3.54% and 3.44%, respectively. As observed with the fluorescence measurements shown in Fig. 1, UV harvesting by AuQDs and energy/electron transfer to plasmonic AuNPs should play an important role in enhancing the device performance. Therefore, light management, namely a fluorescent plasmonic system, should play an important role in developing photovoltaics. By comparing the efficiencies of the AuQD/AuNP-OSCs and AuQD-OSCs (without AuNPs), we can determine how much synergistic enhancement is obtained from each AuQD/AuNP system. The enhancement of the G-AuQD/AuNP-OSCs (*η* = 3.66%) compared to G-AuQD-OSCs (*η* = 3.50%) was found to be 4.6%, while the enhancement of B-AuQD/AuNP-OSCs (*η* = 3.44%) compared to B-AuQD-OSCs (*η* = 3.32%) was 3.6%, and for R-AuQD/AuNP-OSCs (*η* = 3.54%) it was 2.6% compared to R-AuQD-OSCs (*η* = 3.45%). The enhancement of the G-AuQD/AuNP combination was much more than that of the R-AuQD/AuNP combination, indicating that the best synergistic effect was obtained in this system. This is reasonable because the energy transfer, which enhances the localized surface plasmon excitation, can be obtained in the G-AuQD/AuNP system due to the overlap of the wavelength between the fluorescence of G-AuQDs and the localized plasmon peak of AuNPs. For the B-AuQD/AuNP-OSCs, the enhancement was greater than that of the R-AuQD/AuNP-OSCs, although not as high as that for G-AuQD/AuNP-OSCs. This could be due to the significant aggregation of B-AuQDs observed in the AFM images and also due to the reduced energy/electron transfer compared to that of the G-AuQD/AuNP system observed in Fig. 1.

**Table 1:** Photovoltaic parameters of the developed OSCs

Devices	Parameters				
	*J*_sc_ (mA cm^−2^)	*V*_oc_ (V)	FF (%)	PCE (%)	Enhancement (%)
Reference	6.85 ± 0.08	0.59	0.59	3.24	—
AuNPs	7.28 ± 0.05	0.59	0.60	3.42	5.56
B-AuQDs	7.05 ± 0.15	0.59	0.59	3.32	2.47
G-AuQDs	7.33 ± 0.02	0.59	0.60	3.50	8.02
R-AuQDs	7.21 ± 0.07	0.60	0.60	3.45	6.48
B-AuQDs/AuNPs	7.20 ± 0.11	0.59	0.60	3.44	6.17
G-AuQDs/AuNPs	7.61 ± 0.04	0.60	0.60	3.66	13.0
R-AuQDs/AuNPs	7.38 ± 0.05	0.60	0.60	3.54	9.26

**Fig. 3:**
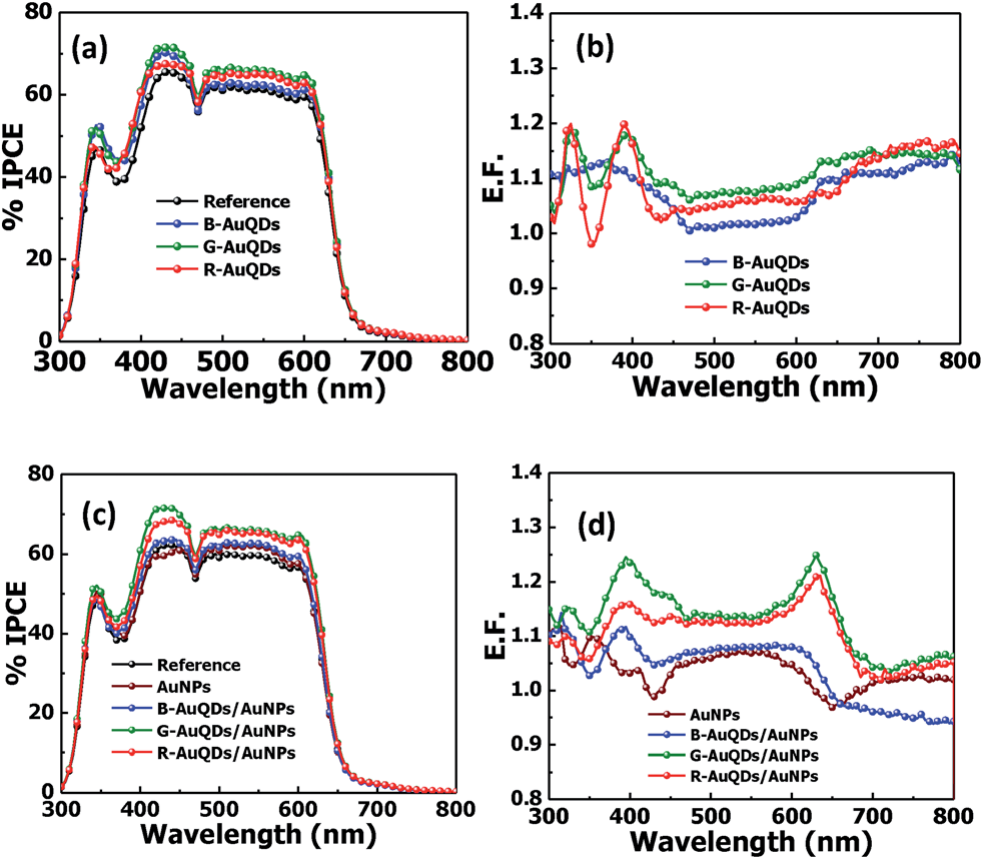
(a) % IPCE and (b) E.F. profiles for the AuQD-only layer system and (c) % IPCE and (d) E.F. profiles for the AuQD/plasmonic AuNP-OSCs.

To study the cooperative effects of AuQDs and AuNPs on the OSC properties, especially photocurrent responses or *J*_sc_, we measured the incident photon-to-current efficiency (IPCE) spectra (incident light wavelength 300–800 nm). The solar cells fabricated with individual AuQDs demonstrated an improvement in the IPCE spectra as shown in Fig. 3(a). (Here, the V-shaped dip at around 470 nm originated from the light source characteristic, which was not related to the device performance.) These devices showed dramatically enhanced IPCE values over a broad wavelength range of 300–800 nm. Incorporating G-AuQDs into OSCs produced the greatest IPCE improvement, comparable to the increase in the *J*_sc_ and PCE values, followed by those of R-AuQDs and B-AuQDs. Additionally, the IPCE enhancement factors (E.F.), obtained by dividing the IPCE values of the developed OSCs by the IPCE values of the reference solar cell, were plotted against the incident light wavelength as presented in Fig. 3(b). This clearly shows enhancement of the IPCE values for all OSCs based on a AuQD layer only. The effect of the AuQD layer on OSCs can be described as follows. The E.F. in the near-UV wavelength region, particularly around 300–500 nm, were induced by absorption by AuQDs within the device. *Via* a quantum effect, these absorbed UV and emitted fluorescence at longer wavelengths, especially in the visible light region, to be absorbed by photoactive materials in the OSCs. Therefore, an increase in the E.F. from 300–500 nm was found, by which AuQDs generated fluorescence in the visible region, which promoted a higher number of photocarriers in the photoactive layer. Over the region 420–680 nm, it was found that G-AuQDs offer the highest improvement by emitting the highest intensity fluorescence, which should be available for absorption by the photoactive layer, and by a plasmonic-like effect associated with the aggregated AuQDs.^25^ With respect to the IPCE in the wavelength range 700–800 nm, R-AuQDs provided the highest enhancement due to their fluorescence. In the region 450–800 nm, the E.F. for B-AuQD-OSCs were lower than those for the other two OSCs, even while presenting strong fluorescence emission. This might be caused by significant aggregation, which generally occurs with small high-surface-area nanoparticles,^31^ thus lowering the photovoltaic properties of our OSCs.

Furthermore, the combination of AuQDs and plasmonic AuNPs in the HTLs of OSCs was studied. Again, the IPCE spectra and E.F. of AuQD/plasmonic AuNP-OSCs were investigated as shown in Fig. 3(c) and (d), respectively. A greater improvement in the IPCEs over a similar wavelength region was observed for all OSCs. Fig. 3(d) presents a larger IPCE enhancement in the G-AuQD/plasmonic AuNP system, which showed the best improvement in the IPCE, which correlated with its *J*–*V* properties. It was found that this combination improved our device over the full wavelength range. In the region from 375–650 nm, the OSC with plasmonic AuNPs incorporated into the HTL showed a lower IPCE enhancement than AuQD/plasmonic AuNP-OSCs, but its IPCE was higher than that of the reference OSC. The G-AuQD/plasmonic AuNP-OSC showed an increased IPCE over the whole wavelength range. In this case, the enhancement of the AuQD/plasmonic AuNP-OSCs could result from a variety of unique properties of AuQDs and AuNPs within the devices. This could be explained mainly according to two phenomena. Firstly, the plasmonic effect (localized plasmon excitation) of AuNPs, which enhanced the light trapping and the optical absorption cross-section of the device, improved the efficiency.^1,30^ Mixing the AuNPs into the PEDOT:PSS or HTL could increase the absorption possibilities of the photoactive layer and also increase the exciton generation rate and probability of exciton dissociation.^32^ Secondly, AuQDs exhibit fluorescence emission that depends on the quantum size effect.^33^ B-AuQD, G-AuQD, and R-AuQD layers not only enhance the light harvesting from the UV region but also generate specific fluorescence emission inside the fabricated devices.^21^ In this study, incorporating a AuQD layer into the plasmonic AuNP devices also improved their efficiency. In particular, the best G-AuQD/plasmonic AuNP-OSC demonstrated UV absorption and generated strong fluorescence emission (a maximum wavelength of *ca.* 525 nm), with energy that matched localized plasmon excitation, facilitating energy transfer from the AuQDs to the plasmonic AuNPs; therefore, this contributed to the largest increase in absorption and photocarrier generation in the P3HT:PCBM active layer.

### Impedance spectroscopy of AuQD/plasmonic AuNP-OSCs

**Fig. 4:**
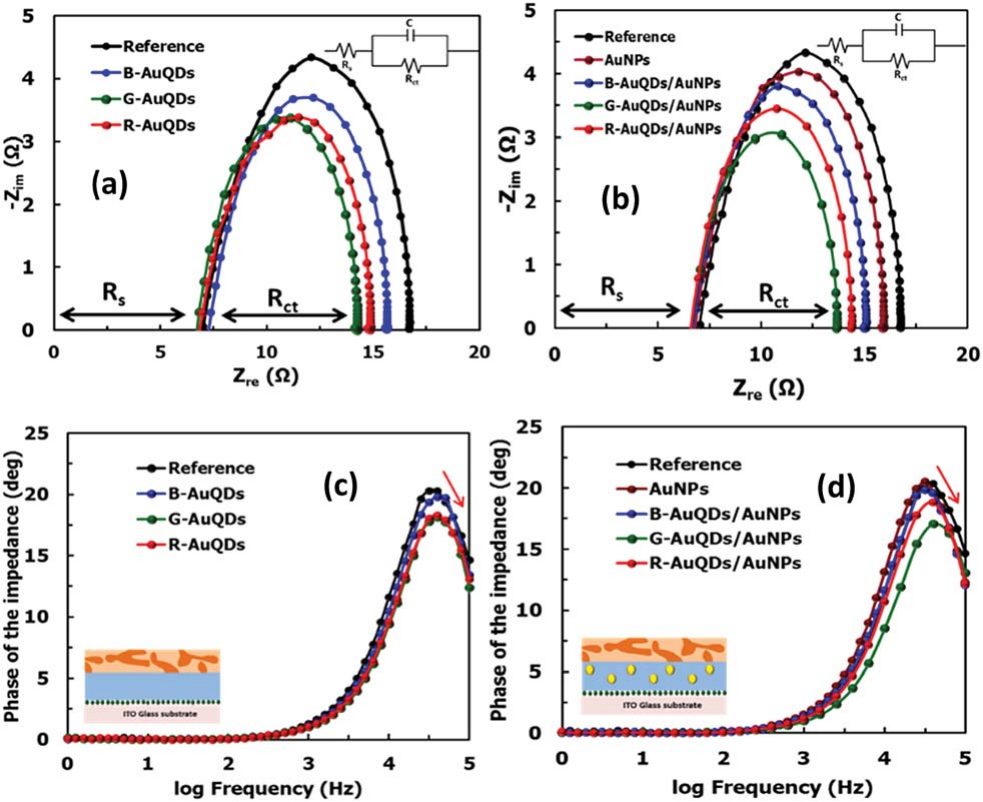
Nyquist plots of the OSCs based on (a) individual AuQD layers and (b) the AuQD/plasmonic AuNP systems under solar light illumination. Bode phase plots of the OSCs based on (c) individual AuQD layers and (d) the AuQD/plasmonic AuNP systems under solar light illumination.

To further study the enhancement effect, electrochemical impedance spectroscopy was used to analyze the internal resistances and carrier transport kinetics in the OSCs.^34,35^ In this measurement, the interfacial properties of the developed OSCs with AuQDs and AuQDs/plasmonic AuNPs were studied. Nyquist plots of the impedance spectra under solar light illumination for the developed OSCs, including AuQD and AuQD/plasmonic AuNP systems, are shown in Fig. 4(a) and (b), respectively. All the devices exhibited a single semicircular curve in their Nyquist plots, and significant differences among the impedance spectra of each device were observed. Simple equivalent circuit models are shown in the insets of Fig. 4(a) and (b). In this model, the *R*_s_ value or contact resistance in series represents the resistive loss in the ITO and PEDOT:PSS, corresponding to the intersection of the semicircles.^30^ We found that the *R*_s_ values of both AuQD and AuQD/plasmonic AuNP devices were slightly smaller than that of the reference cell. This indicated that the interfacial contact resistance of the fabricated devices was decreased by insertion of the AuQD layer, loading AuNPs into the PEDOT:PSS film, or combining AuQD and AuNP:PEDOT:PSS layers. On the other hand, the constant phase element in parallel with the charge transfer resistance (*R*_ct_) corresponds to the capacitance, which is used to describe the distribution of carrier relaxation time at the active layer.^36^ The *R*_ct_ values of the fabricated plasmonic solar cells decreased as a result of incorporation of the plasmonic layers. The *R*_ct_ values of the devices were derived as follows: 9.8 Ω for the cells with no plasmonic nanoparticles, 8.9 Ω for those with a AuNP:PEDOT:PSS layer only, 8.3 Ω for those with a B-AuQD layer only, 7.9 Ω for those with a R-AuQD layer only, and 7.3 Ω for those with a G-AuQD layer only. Interestingly, incorporating both AuNP:PEDOT:PSS and AuQD layers into OSCs resulted in lower *R*_ct_ values compared to those of individual systems, which were 8.1, 7.4, and 6.7 Ω for AuNP:PEDOT:PSS with B-AuQD, R-AuQD, and G-AuQD systems, respectively. This indicates that introducing AuNPs into the hole-transport layer (HTL) and a layer of AuQDs into OSCs could enhance the charge transfer.^30^ Adding a AuQD layer could increase the number of photocarriers in the device *via* its fluorescence properties, which is consistent with the results obtained from *J*–*V* measurements. These photogenerated carriers in the P3HT:PCBM layer should result from the absorption of UV and visible light fluorescence emission in the AuQD layer. Furthermore, the combination of AuQDs and AuNPs further increased the photocarriers. This could be due to an enhanced localized plasmon field at the AuNPs caused by the fluorescence emission of AuQDs and energy/electron transfer from the AuQDs to AuNPs, which corresponded to the fluorescence quenching shown in Fig. 1. Frequency peaks of the Bode phase plots for AuQD- and AuQD/plasmonic AuNP-OSCs are shown in Fig. 4(c) and (d), respectively. The frequency peaks are related to the electron lifetime in the devices.^9,34,37^ It could be seen that after adding a AuQD layer to the devices, the frequency peaks slightly shifted to higher frequency in comparison with that of the reference cell. Similarly, the OSCs with AuQD/plasmonic AuNP systems exhibited higher frequency peaks compared to that of a cell with only AuNPs. The average electron lifetimes in organic solar cells can be investigated using impedance spectroscopy.^36,38^ The characteristic frequency peak of each OSC in Bode phase plots was employed to calculate the average electron lifetime (*τ*_avg_).^15,39^ The average carrier lifetimes are summarized in Table S21 (ESI).† The results are not significantly different, suggesting that AuNP:PEDOT:PSS and/or AuQD layers might not significantly affect the carrier lifetime. Therefore, modification of the OSC configurations with the AuQD/AuNP complex improved the device performance mainly by the enhanced photocarrier generation due to the synergistic effect of fluorescence emission and enhanced plasmonic properties *via* energy/electron transfer from AuQDs to AuNPs.

## Conclusions

We successfully designed an efficient AuQD/plasmonic AuNP system to manage light harvesting to enhance OSCs. A AuQD layer with green fluorescence emission and a AuNP:PEDOT:PSS HTL with localized plasmon excitation achieved the best light harvesting in OSCs. A G-AuQD/AuNP complex system exhibited enhanced photovoltaic performance (a *J*_sc_ of 7.61 mA cm^−2^ and a PCE of up to 3.66% (13% improvement)), compared to reference OSCs. AuQDs could broaden light harvesting in the UV region and emit light in the visible region, which could be absorbed by the active layer as well as inducing energy/electron transfer to the plasmonic AuNPs, resulting in increased light harvesting in OSCs. Hence, the AuQD/AuNP complex system designed for OSCs has the potential to synergistically enhance OSC performance. Our strategy for light manipulation in OSCs using AuQDs and AuNPs is promising and could be applied to the development of other types of solar cells.

## Conflicts of interest

There are no conflicts to declare.

## Supplementary Material

NA-001-C8NA00119G-s001
