# Supplementary material for: Investigation of a gold quantum dot/plasmonic gold nanoparticle system for improvement of organic solar cells
Source: Nanoscale Adv. 2018 Nov 8;1(2):792–8. doi: 10.1039/c8na00119g (PMC9473205; doi:10.1039/c8na00119g)
Supplement: NA-001-C8NA00119G-s001 [file NA-001-C8NA00119G-s001.pdf]

Electronic Supplementary Material (ESI) for Nanoscale Advances.
This journal is © The Royal Society of Chemistry 2018

Electronic Supplementary Information

Investigation of Gold Quantum Dot/Plasmonic Gold Nanoparticle System for Improvement of Organic Solar Cells

Sopit Phetsang,[ab] Apichat Phengdaam,[c] Chutiparn Lertvachirapaiboon,[a] Ryousuke Ishikawa,[a] Kazunari Shinbo,[a] Keizo Kato,[a] Pitchaya Mungkornasawakul,[bd] Kontad Ounnunkad*[be] and Akira Baba*[a]

[a]Graduate School of Science and Technology, Niigata University, 8050 Ikarashi-2-nocho, Nishi-ku, Niigata 950-2181, Japan.

E-mail: ababa@eng.niigata-u.ac.jp

[b]Department of Chemistry and Center of Excellence for Innovation in Chemistry (PERCH-CIC), Faculty of Science, Chiang Mai University, Chiang Mai 50200, Thailand.

E-mail: kontad.ounnunkad@cmu.ac.th

[c]Department of Chemistry, Faculty of Science, Prince of Songkla University, Hat Yai, Songkla, 90110, Thailand.

[d]Environmental Science Program, Faculty of Science, Chiang Mai University, Chiang Mai 50200, Thailand.

[e]Center of Excellence in Materials Science and Technology, Chiang Mai University, Chiang Mai 50200, Thailand

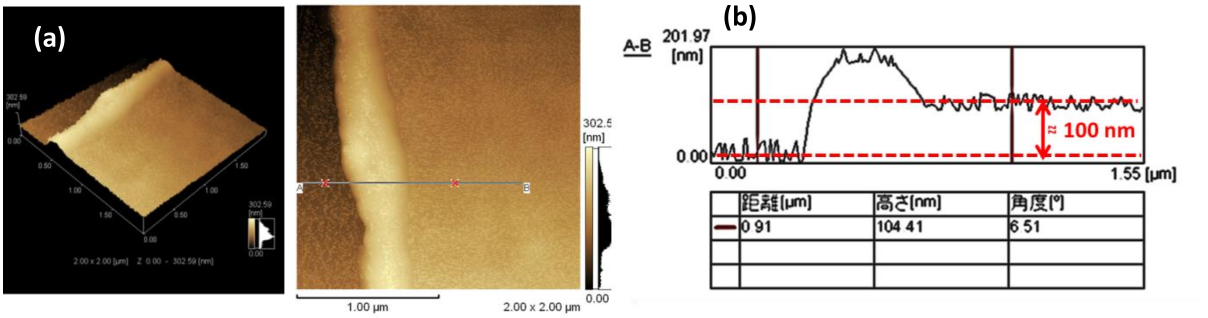

Figure S1 (a) AFM image and (b) cross-sectional profile of a AuQD/PEDOT:PSS film on a substrate, indicating the thickness of the film (100 nm).

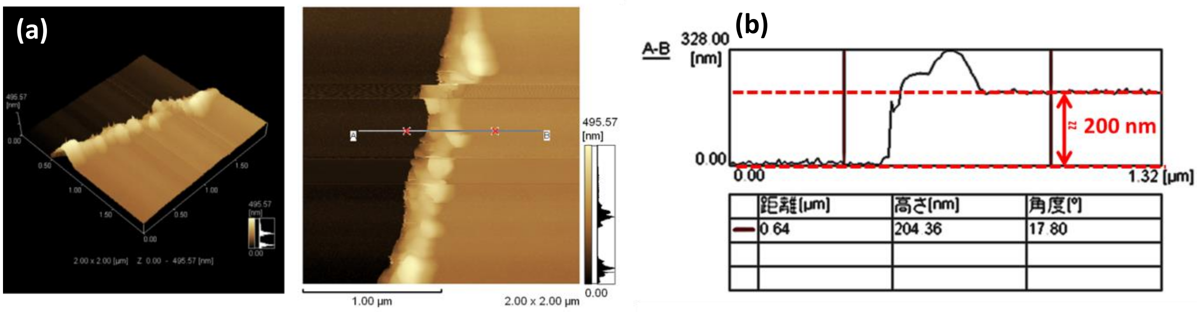

Figure S2 (a) AFM image and (b) cross-sectional profile of a AuQD/PEDOT:PSS/P3HT:PCBM film on a substrate, indicating the thickness of the film (200 nm).

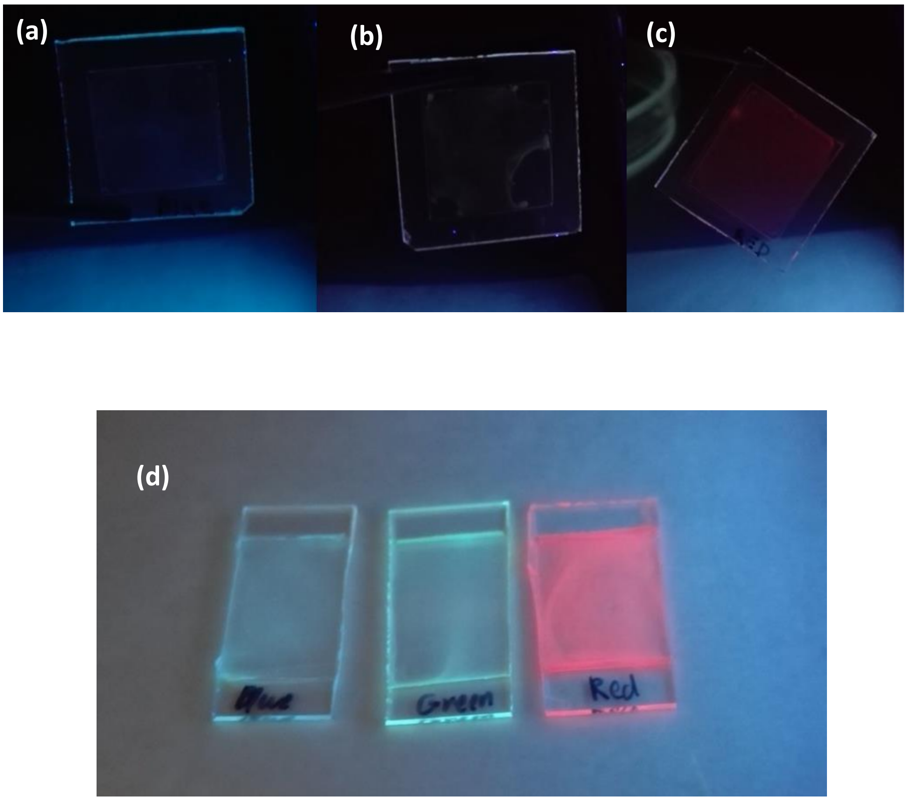

Figure S3 Fluorescence emission characteristics of AuQD films on glass substrates under UV illumination. (a) B-AuQD film, (b) G-AuQD film, and (c) R-AuQD film deposited by spin-coating method, (d) drop-casted 3 types of AuQD films.

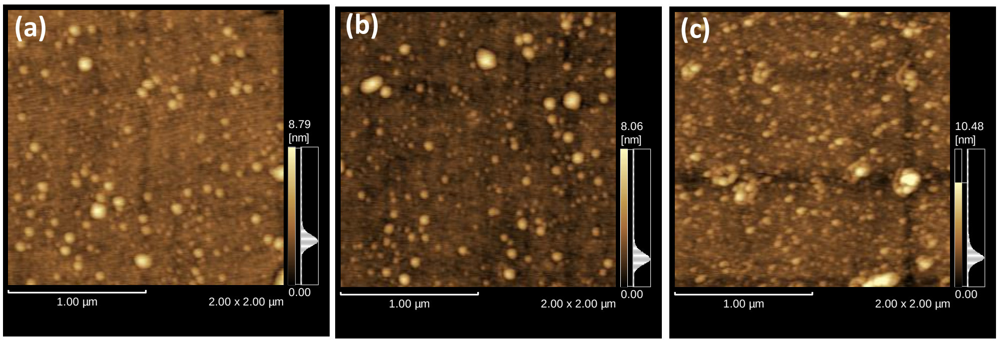

Figure S4 AFM images of AuQD films after direct spin-coating on glass substrates, (a) B-AuQDs, (b) G-AuQDs, and (c) R-AuQDs at a concentration of 0.0050 mM

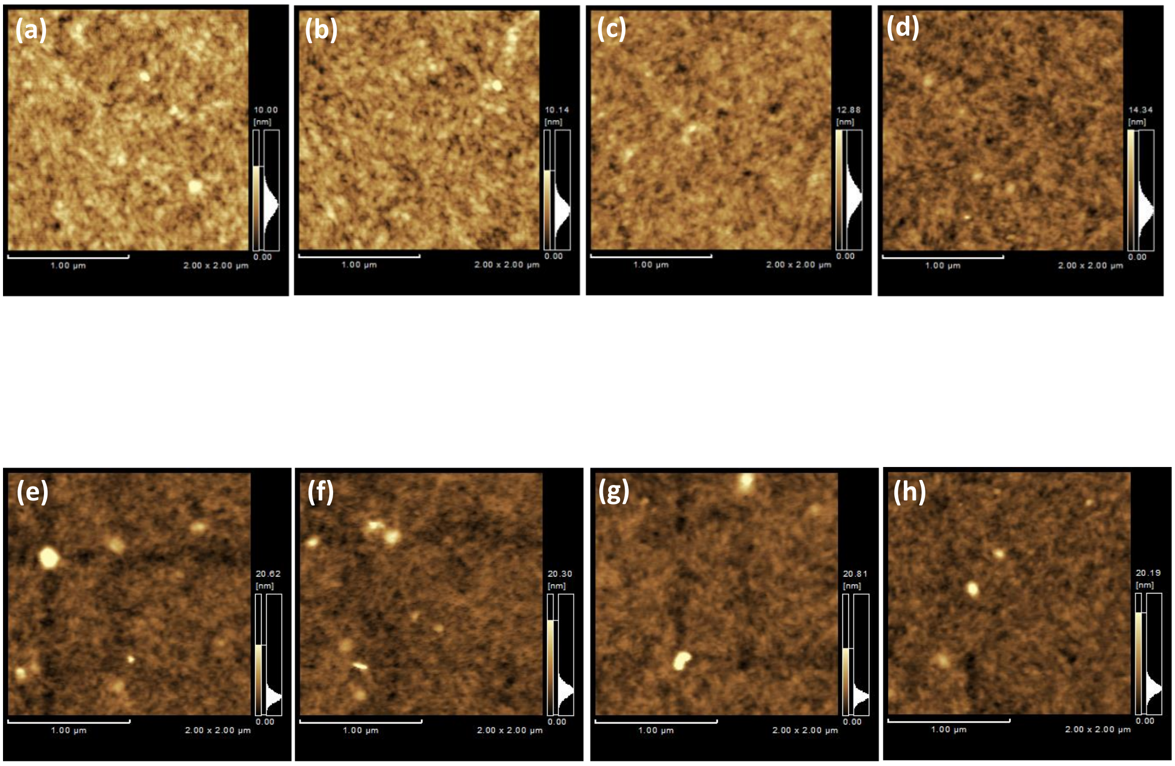

Figure S5 Surface morphology of PEDOT:PSS films on (a) ITO substrate (reference cell), (b) B-AuQD layer, (c) G-AuQD layer, (d) R-AuQD layer, and of PEDOT:PSS:AuNP films on (e) ITO substrate, (f) B-AuQD layer, (g) G-AuQD layer, and (h) R-AuQD layer.

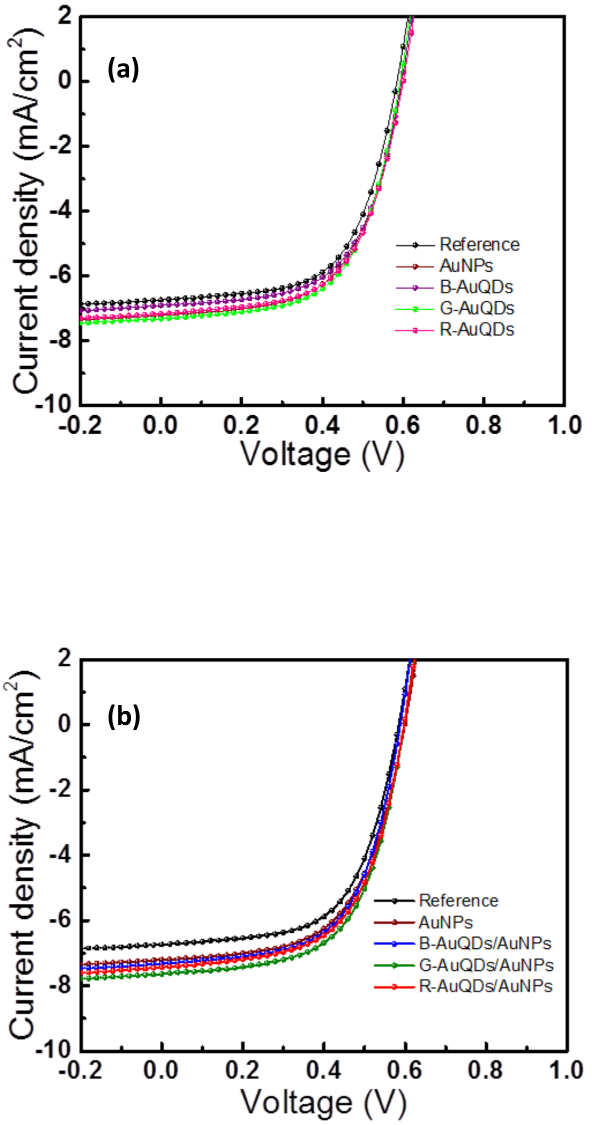

Figure S6 (a) *J-V* characteristics of the AuQD-loaded OSCs (b) *J-V* characteristics of the AuQD/plasmonic AuNP OSCs compared with those of the reference cell.

Table S1 Optimization of AuQD contents in the developed OSCs

Devices	J_{sc} (mA/cm^2)	V_{oc} (V)	FF (%)	PCE (%)
**Reference**	6.85	0.59	0.59	3.24
**AuNPs 0.1 mM**	7.28	0.59	0.60	3.42
**B-AuQDs/AuNPs**				
0.0100 mM	6.83	0.58	0.61	3.28
0.0050 mM	7.20	0.59	0.60	3.44
0.0025 mM	7.11	0.59	0.62	3.43
0.0012 mM	7.08	0.59	0.61	3.43
**G-AuQDs/AuNPs**				
0.0100 mM	7.00	0.58	0.60	3.37
0.0050 mM	7.61	0.60	0.60	3.66
0.0025 mM	7.48	0.59	0.61	3.59
0.0012 mM	7.11	0.59	0.61	3.45
**R-AuQDs/AuNPs**				
0.0100 mM	7.20	0.59	0.60	3.46
0.0050 mM	7.38	0.60	0.60	3.54
0.0025 mM	7.14	0.58	0.61	3.42
0.0012 mM	7.06	0.59	0.61	3.41

Table S2 Power conversion efficiency (PCE), short-circuit current density (J_{sc}), average electron lifetime (τ_{avg}), and maximum frequency (f_{max}) of fabricated OSCs

Devices	PCE (%)	J_{SC} (mA/cm^2)	τ_{avg} (µs)	f_{max} (kHz)
Reference	3.24±0.03	6.85±0.08	5.05	31.62
AuNPs	3.42±0.02	7.28±0.05	5.05	31.62
B-AuQDs	3.32±0.02	7.05±0.15	4.48	35.48
G-AuQDs	3.50±0.01	7.33±0.02	4.00	39.81
R-AuQDs	3.45±0.04	7.21±0.07	3.56	44.67
B-AuQDs/AuNPs	3.44±0.04	7.20±0.11	4.48	35.48
G-AuQDs/AuNPs	3.66±0.03	7.61±0.04	4.00	39.81
R-AuQDs/AuNPs	3.54±0.01	7.38±0.05	4.00	39.81
